# Bioguided Fractionation of Local Plants against Matrix Metalloproteinase9 and Its Cytotoxicity against Breast Cancer Cell Models: In Silico and In Vitro Study (Part II)

**DOI:** 10.3390/molecules26051464

**Published:** 2021-03-08

**Authors:** Maywan Hariono, Rollando Rollando, I Yoga, Abraham Harjono, Alfonsus Suryodanindro, Michael Yanuar, Thomas Gonzaga, Zet Parabang, Pandu Hariyono, Rifki Febriansah, Adi Hermawansyah, Wahyuning Setyani, Habibah Wahab

**Affiliations:** 1 Drug Discovery Student Club, Faculty of Pharmacy, Campus III, Sanata Dharma University, Paingan, Maguwoharjo, Depok, Sleman, Yogyakarta 55282, Indonesia; kresnayoga65@gmail.com (I.Y.); abrahamoliver29@gmail.com (A.H.); alfonsusivan@gmail.com (A.S.); gregoriusrest@gmail.com (M.Y.); davidgonzaga017@gmail.com (T.G.); zetariparabang@gmail.com (Z.P.); michaelpandu99@gmail.com (P.H.); wahyuningsetyani@usd.ac.id (W.S.); 2 Bachelor in Pharmacy Program, Faculty of Science and Technology, Ma Chung University, Malang 65151, Indonesia; ro.llando@machung.ac.id; 3 School of Pharmacy, Faculty of Medicine and Health Sciences, Universitas Muhammadiyah Yogyakarta, Kasihan, Bantul, Yogyakarta 55183, Indonesia; rifki.febriansah@umy.ac.id; 4 Cell Culture Laboratory, Faculty of Medicine and Health Sciences, Universitas Muhammadiyah Yogyakarta, Kasihan, Bantul, Yogyakarta 55183, Indonesia; hermawansyah_adi@yahoo.com; 5 Pharmaceutical Technology Department, School of Pharmaceutical Sciences and USM-RIKEN Centre for Ageing Science (URICAS), Universiti Sains Malaysia, Minden, Pulau Pinang 11800, Malaysia; habibahw@usm.my

**Keywords:** MMP9, PEX9, cancer, bioguided, fractionation, ageratum, screening, in silico, in vitro

## Abstract

In our previous work, the partitions (1 mg/mL) of *Ageratum conyzoides* (AC) aerial parts and *Ixora coccinea* (IC) leaves showed inhibitions of 94% and 96%, respectively, whereas their fractions showed IC_50_ 43 and 116 µg/mL, respectively, toward Matrix Metalloproteinase9 (MMP9), an enzyme that catalyzes a proteolysis of extracellular matrix. In this present study, we performed IC_50_ determinations for AC *n*-hexane, IC *n*-hexane, and IC ethylacetate partitions, followed by the cytotoxicity study of individual partitions against MDA-MB-231, 4T1, T47D, MCF7, and Vero cell lines. Successive fractionations from AC *n*-hexane and IC ethylacetate partitions led to the isolation of two compounds, oxytetracycline (OTC) and dioctyl phthalate (DOP). The result showed that AC *n*-hexane, IC *n*-hexane, and IC ethylacetate partitions inhibit MMP9 with their respective IC_50_ as follows: 246.1 µg/mL, 5.66 µg/mL, and 2.75 × 10^−2^ µg/mL. Toward MDA-MB-231, 4T1, T47D, and MCF7, AC *n*-hexane demonstrated IC_50_ 2.05, 265, 109.70, and 2.11 µg/mL, respectively, whereas IC ethylacetate showed IC_50_ 1.92, 57.5, 371.5, and 2.01 µg/mL, respectively. The inhibitions toward MMP9 by OTC were indicated by its IC_50_ 18.69 µM, whereas DOP was inactive. A molecular docking study suggested that OTC prefers to bind to PEX9 rather than its catalytic domain. Against 4T1, OTC showed inhibition with IC_50_ 414.20 µM. In conclusion, this study furtherly supports the previous finding that AC and IC are two herbals with potential to be developed as triple-negative anti-breast cancer agents.

## 1. Introduction

Cancer is the second leading cause of death in the world with 9.6 million deaths in 2018, with one-in-six mortality caused by this disease [1]. In Asia, approximately 8.75 million new cases and 5.5 million deaths were reported in that same year. In our country (Indonesia), almost 350,000 new cases and 208,000 deaths were reported due to cancer [2]. Triple-negative breast cancer has especially attracted more attention due to the high mortality in females [3]. The high expression of the matrix metalloproteinase9 (MMP9) protein is one of the indicators during diagnosis [4,5]. Although drug discovery targeting this protein is desirable, a potent MMP9 inhibitor is usually limited by its adverse reaction to drugs, such as musculoskeletal syndromes [6,7].

In our previous work, virtual screening for PEX9 inhibitors was performed to identify the top plants that have potential selective anticancer agents from herbals. Twenty of 200 compounds from our in-house database were selected as top hits based on the rank of binding energy toward PEX9. These 20 hit compounds were linked to the plants from which they were reported to be isolated. The hit plants were prepared in a methanolic extract to be subjected to a step-by-step in vitro screening against MMP9. The samples were from its crude extract, partitioned until fractioned, and tested using Fluorescence Resonance Energy Transfer (FRET)-based assay in vitro. The partitions were prepared by sub-extracting the methanolic extracts using *n*-hexane, ethylacetate, *n*-butanol, and water to represent the nonpolar to polar part of the crude extracts. The fractions were collected from the selected partition using column chromatography, having the best inhibition percentage toward MMP9. Interestingly, six crude extracts demonstrated inhibition toward MMP9 with the IC_50_ within 24 to 823 µg/mL [8].

The investigation was then focused on the potency of two crude extracts in vitro, i.e., *Ageratum conyzoides* (AC) and *Ixora coccinea* (IC), which, on one hand, demonstrated inhibition toward MMP9 with an IC_50_ of 64 and 82 µg/mL, respectively. On the other hand, interestingly, AC crude extract showed inhibition toward 4T1 (IC_50_ 22 µg/mL) and T47D (IC_50_ 163 µg/mL), whereas IC crude extract had a weaker inhibition toward 4T1 (IC_50_ 270 µg/mL) and T47D (IC_50_ 2200 µg/mL). Further exploration of these two plants was carried out by sub-extracting their crude extracts to have four types of partition, namely, *n*-hexane, ethylacetate, *n*-butanol, and water partitions. The four partitions of the individual plants (AC and IC) were then tested for their inhibition against MMP9 in vitro, with the resulting inhibitions as follows: AC (*n*-hexane = 94%; ethylacetate = 97%; *n*-butanol = 97%; and water = 94%) and IC (*n*-hexane = 94%; ethylacetate = 86%; *n*-butanol = 93%; and water = 48%), supporting the previous results that these two plants have great potential to be developed further in search for anticancer agents from herbals [8].

**Figure 1:**
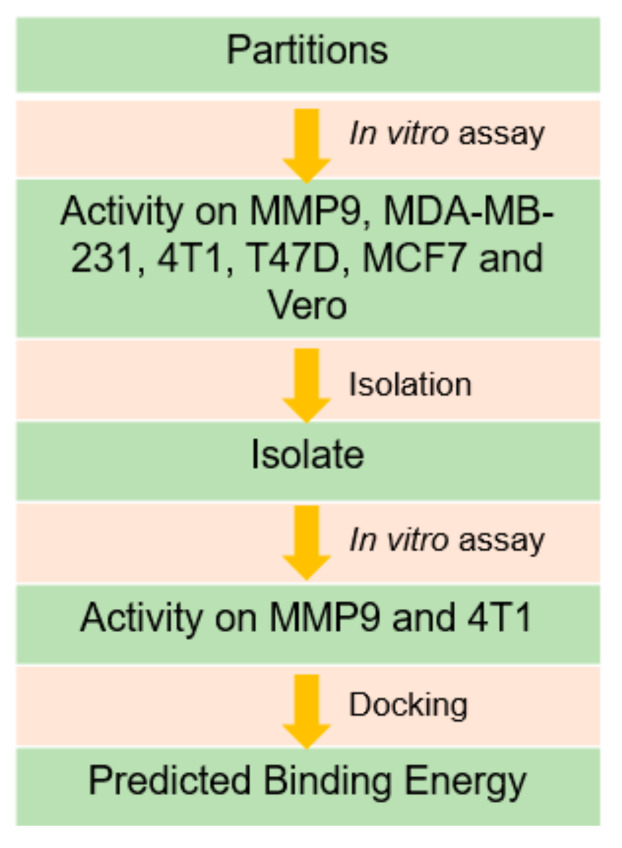
The flow chart of the studies in bioguided fractionation of local plants to identify Matrix Metalloproteinase9 (MMP9) inhibitors and breast cancer cytotoxic agents.

As a continuation, in this present study, the partitions of *n*-hexane of AC and *n*-hexane and ethylacetate of IC were selected for the determination of IC_50_ against MMP9 as well as against four types of cancer cell lines, namely, MDA-MB-231, 4T1, T47D, and MCF7. The safety index was also determined against Vero cells. To answer our curiosity about which compound is responsible for the biological activity in inhibiting both MMP9 and cancerous cell lines, compound isolation from *n*-hexane of AC and ethylacetate of IC partitions was carried out. Figure 1 presents the flow chart for this study. MDA-MB-231 and 4T1 represented highly metastatic cancer cell models (triple negative types), whereas T47D and MCF7 were representative of non-metastatic cancer cell models (luminal types). Theoretically, the samples should have been more active with MDA-MB-231 and 4T1 than T47D and MCF7, if the samples were active against MMP9. In conjunction, the isolated compounds from the sample partitions should also have had a higher affinity to the hemopexin domain of MMP9 rather than its catalytic site using in silico docking. This would support our previous results that by targeting PEX9, it would hypothetically result in more selective cancer cell antiproliferation rather than the normal cell cytotoxicity since the PEX9 domain has been demonstrated to be a more selective target than its catalytic site.

## 2. Results

### 2.1. MMP9 In Vitro Assay

**Figure 2:**
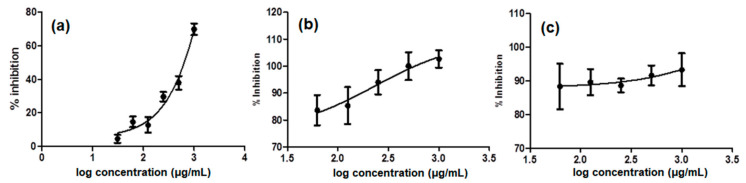
The drug dose-dependent curves of (**a**) *Ageratum conyzoides* (AC) *n*-hexane (IC_50_ 246.1 µg/mL; R^2^ 0.9620), (**b**) *Ixora coccinea* (IC) *n*-hexane (IC_50_ 5.66 µg/mL; R^2^ 0.5036), and (**c**) IC ethylacetate (IC_50_ 2.75 × 10^−2^ µg/mL; R^2^ 0.0754) as the results of Fluorescence Resonance Energy Transfer (FRET)-based assay toward MMP9. *N*-isobutyl-*N*-(4-methoxyphenylsulfonyl)-glycyl hydroxamic acid (NNGH) was used as the positive control (IC_50_ 47.8 nM). The bars show the standard error of the triplicate assay as previously described.

In the first investigation by IC_50_ determination of the three selected partitions against MMP9, IC ethylacetate showed potent activity with an IC_50_ of 2.75 × 10^−2^ µg/mL (Figure 2c). As we can see from the drug dose-dependent curve, IC ethylacetate showed nearly 90% inhibition in all concentrations tested (62.5 to 1000 µg/mL), hence an almost flat curve. The second best activity was performed by IC’s *n*-hexane, with an IC_50_ of 5.66 µg/mL (Figure 2b). This partition demonstrated 83% inhibition at the lowest concentration, i.e., 62.5 µg/mL, with the activity increasing as the concentration increased up to 1000 µg/mL. However, this trend was different in the case of AC *n*-hexane (Figure 2a), where low MMP9 inhibition (10%) started to be observed at 62.5 µg/mL and increased only up to 68% as the concentration reached 1000 µg/mL. An IC_50_ of AC *n*-hexane was obtained at 246.1 µg/mL, confirming its moderate activity as an MMP9 inhibitor.

### 2.2. Cytotoxicity In Vitro Assay of the Partitions

The three partitions were further evaluated for their cytotoxicity against four types of breast cancer cell models. MDA-MB-231 is a triple-negative cell model from humans in the last stage of breast cancer [9], whereas 4T1 is a highly metastasized cell model from the mouse mammary gland. These cancer cell models are commonly studied due to the high expression of MMP9 during metastasis [10]. T47D and MCF7 are two human hormone-dependent breast cancer cell lines that are widely used as experimental models for in vitro and in vivo (tumor xenografts) breast cancer studies [11]. Table 1 presents the results of the in vitro cytotoxicity assay of the three partitions against MDA-MB-231, 4T1, T47D, MCF7, and Vero cell proliferation. Unfortunately, the cytotoxicity effects against MDA-MB-231 and MCF7 of IC *n*-hexane could not be evaluated due to its high contaminations. The IC *n*-hexane treatment in MDA-MB-231 and MCF7, upon continuous observation, showed the cell to be different in size, round and growing fast, which could have already been contaminated by mycoplasma [12], therefore, the evaluation of this sample could not proceed. This work should be repeated to confirm the cell condition, but due to our limitation, at the moment, we only can suggest this work for future study.

As observed in Table 1, AC *n*-hexane showed high cytotoxicity against MDA-MB-231 and MCF7 with respective IC_50_ of 2.05 and 2.11 µg/mL. In contrast, this partition showed a low cytotoxic effect against 4T1 and T47D with respective IC_50_ of 265 and 109.70 µg/mL. IC *n*-hexane was observed to have a cytotoxic effect against 4T1 stronger than T47D, as shown by respective IC_50_ of 225.5 and 1320 µg/mL. At the same concentration series, IC ethylacetate also had a strong cytotoxic effect against MDA-MB-231 and MCF7 with respective IC_50_ of 1.92 and 2.01 µg/mL. This partition demonstrated a stronger cytotoxic effect against 4T1 than AC *n*-hexane and IC *n*-hexane with its IC_50_ of 57.5 µg/mL. However, its cytotoxic effect against T47D was in between AC *n*-hexane’s and IC *n*-hexane’s IC_50_. Interestingly, these three partitions had stronger cytotoxic effects against 4T1 than doxorubicin, which was used as the positive control (IC_50_ 388.4 µg/mL) in this study. In contrast, doxorubicin showed stronger cytotoxicity (IC_50_ 5.13 µg/mL) against T47D than the three partitions.

The cytotoxicity of the three partitions against the normal cell line (Vero) was also determined. Among these partitions, IC *n*-hexane demonstrated the highest IC_50_ (Table 1), describing its lowest toxic potency to the normal cell (Table 2). However, when compared to the T47D cytotoxicity profile, the safety index (SI) was very low (0.74), defining its non-selective effect when it was applied to a T47D cell. Other low SI values were also observed in AC *n*-hexane in 4T1 and T47D, and in IC ethylacetate in T47D. It is known that an SI value of less than 2.0 indicates the general toxicity of the compound [13]. Therefore, according to the criteria, AC *n*-hexane and IC ethylacetate had high selectivities when they were applied to the MDA-MB-231 (SI 105.95 and 223.69, respectively) and MCF7 (SI 102.93 and 213.68, respectively). Moreover, although they were not as selective against MDA-MB-231 and MCF7, IC *n*-hexane and IC ethylacetate had high selectivities toward 4T1 with respective SIs of 4.31 and 7.47. Doxorubicin as the anticancer drug had a poor SI (0.18) against 4T1, but in contrast, this drug showed much better selectivity (13.56) than the other treatments against T47D.

**Figure 3:**
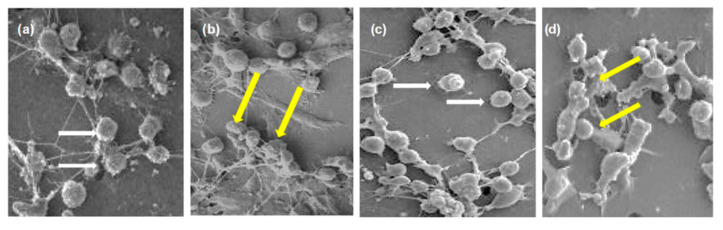
The cell morphologies after and before treatments using AC *n*-hexane as follows: (**a**) untreated MDA-MB-231, (**b**) treated MDA-MB-231, (**c**) untreated MCF7, and (**d**) treated MCF7. The white arrows indicate the normal living cell, whereas the yellow arrows indicate the cell morphology changing.

Figure 3 illustrates the changes in MDA-MB-231 and MCF7 cell morphology after the treatment using AC *n*-hexane from a scanning electron microscope (SEM). The treatment caused the cells to be shrunken and rounded, with some blebbing on the cell membrane, and the border between cells became very thin. In contrast, this condition was not seen in the untreated cells. This deformation could have been caused by the disconnection of the cytoskeleton, and the protein, having a role in the cell connection, may not have polymerized, leading to the detachment of the cells while forming a rounded lipid membrane. On the other hand, there was a reduction in cell viability as well as density as higher concentrations of AC *n*-hexane were used in the treatment. The shrinking cell indicated its starting point that led to the death of the cells. 

**Figure 4:**
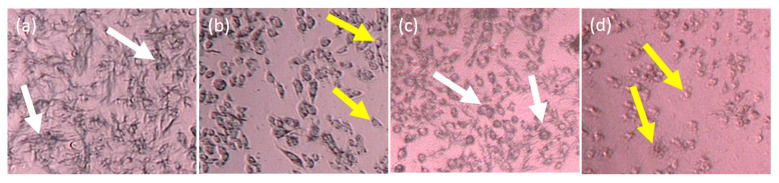
The cell morphologies after and before treatment with IC *n*-hexane as follows: (**a**) untreated T47D, (**b**) treated T47D, (**c**) untreated 4T1, and (**d**) treated 4T1. The white arrows indicate the normal living cells that form formazan crystals whereas the yellow arrows indicate the dead cells.

In the 2D form, the cell morphology of T47D and 4T1 post-treated with IC *n*-hexane partition showed less formation of formazan crystals than their negative control. This indicates that the dead cells after treatment with IC *n*-hexane partition were due to its cytotoxic activity against the cancer cells. The 4T1 cell was characterized by a type E epitel with a crowded cellular distance and resistant toward 6-thioguanine [14], whereas T47D showed a tightly cohesive cobblestone appearance [15]. Figure 4 illustrates the cell morphologies of T47D and 4T1 after IC *n*-hexane treatment.

**Figure 5:**
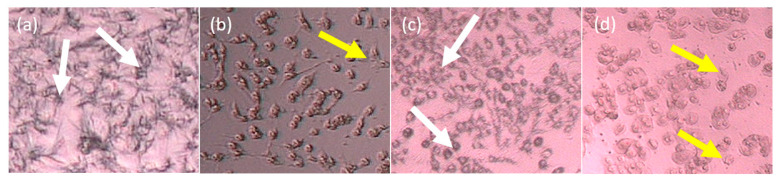
The cell morphologies after and before treatment with IC ethylacetate as follows: (**a**) untreated T47D, (**b**) treated T47D, (**c**) untreated 4T1, and (**d**) treated 4T1. The white arrows indicate the normal living cells that form formazan crystals whereas the yellow arrows indicate the dead cells.

The IC ethylacetate partition also demonstrated its capability to stop the proliferation of T47D and 4T1 cells during MTT assay. The treated cells suffered a random cell deformation after treatment. In terms of number, these were counted to be much less. In general, cells proliferate into high crowds in the media, therefore, a decrease in cell numbers is one of the indicators that the proliferation is inhibited [16]. The T47D living cells after treatment became darker (formazan crystal), whereas the dead cells showed a random deformation and were colorless. Figure 5 illustrates the cell morphologies of T47D and 4T1 after IC ethylacetate partition before and after treatment with the IC ethylacetate partition.

**Figure 6:**
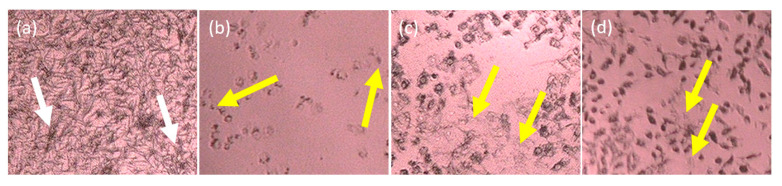
The Vero cell morphologies after and before treatment with three partitions as follows: (**a**) untreated cell, (**b**) AC *n*-hexane, (**c**) IC *n*-hexane, and (**d**) IC ethylacetate. The white arrows indicate the normal living cells that form formazan crystals whereas the yellow arrows indicate the dead cells.

In the Vero cell, the treatment using three partitions showed different effects. AC *n*-hexane partition was more toxic to the Vero cells compared to IC *n*-hexane as well as IC ethylacetate partitions. The number of formazan crystals in this partition was less than the two other partitions, reflecting less cell ability to survive against AC *n*-hexane treatment. The Vero cell is characterized by its fibroblast-like shape, which is elongated with a little cytoplasmic granulation [17]. Figure 6 illustrates the cell morphologies of Vero cells after AC *n*-hexane, IC *n*-hexane, and IC ethylacetate partition treatment.

### 2.3. Isolation and In Vitro Assay

**Figure 7:**
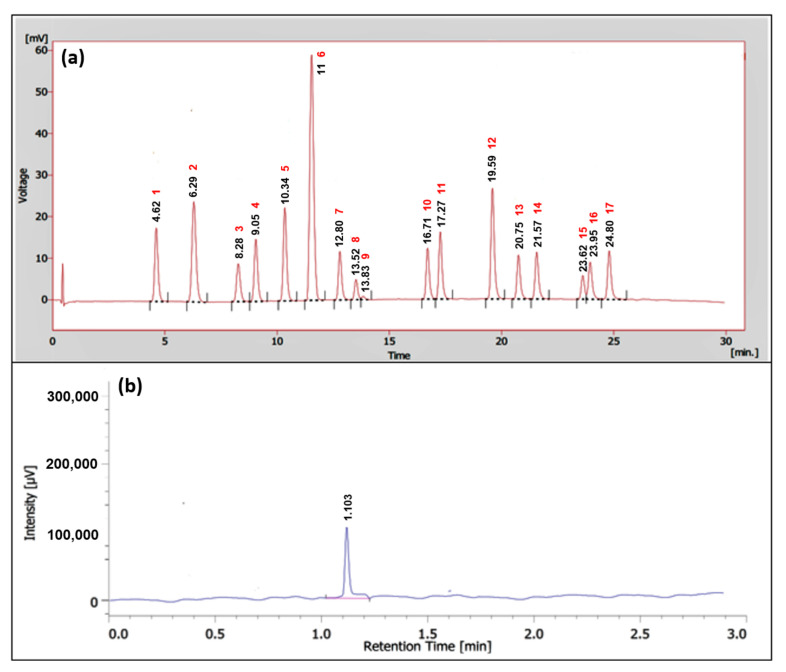
High Performance Liquid Chromatography (HPLC) chromatogram of (**a**) a fraction of AC *n*-hexane and (**b**) its isolated compound.

**Figure 8:**
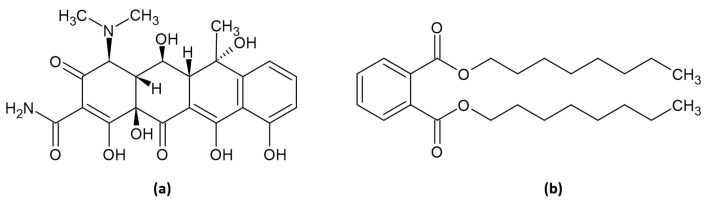
The chemical structures of (**a**) oxytetracyline (OTC) and (**b**) dioctyl phthalate (DOP) isolated from AC *n*-hexane and IC ethylacetate partitions, respectively.

Compound isolation from the two active partitions i.e., AC *n*-hexane and IC ethylacetate against both enzymatic and cellular assay, was successfully conducted. The *n*-hexane AC was fractionated into an *n*-hexane-ethylacetate fraction using conventional column chromatography, and then followed by the isolation of a single compound using preparative- high-performance liquid chromatography (prep-HPLC). As depicted in Figure 7a, there were 17 peaks resolved, implying that there were 17 compounds present in the fraction. One compound with Rt of 11.00 min and area of 28.31% was selected to be a part of the fraction. The purity of the isolated compound was then confirmed by re-running the HPLC and showing the single peak at 1.103 min (Figure 7b). The structural characterization using NMR, FTIR, and GC-MS indicated that the isolated compound from AC *n*-hexane was oxytetracycline (OTC) (Figure 8a).

On the other hand, the IC ethylacetate was also proceeded for fractionation using a conventional column chromatography and was further subjected to single compound isolation using preparative thin-layer chromatography (prep-TLC). Based on the NMR, FTIR, and GC-MS, the single compound from IC ethylacetate was characterized as dioctyl phthalate (DOP) (Figure 8b).

The structural characterizations of OTC and DOP are as follows: (4S,4aR,5S,5aR,6S,12aS)-4-(dimethylamino)-3,5,6,10,12,12a-hexahydroxy-6-methyl-1,11-dioxo-1,4,4a,5,5a,6,11,12a-octahydrotetracene-2-carboxamide (oxytetracycline; OTC); orange powder; yield 5%; m.p. > 300 °C (decomposes); FTIR (cm^−1^) 3374 (OH), 1619 (C=O), 1583 (aromatic C=C), 1455 (C-N), 1177 (C-O); ^1^H-NMR (500 MHz, CDCl_3_) δ 1.95 (s, CH_3_), 2.0 (s, OH), 2.16 (s, CH_3_), 2.20 (t, J = 8 Hz, CH), 2.48 (d, J = 7.5 Hz, CH), 3.13 (t, J = 3 Hz, CH), 3.47 (s, CH_3_), 5.33 (s, OH), 6.12 (dd, J = 37.5 Hz, aromatic), 6.96 (dd, J = 3–7.5 Hz, aromatic), 6.97 (td, J = 3–7 Hz, aromatic); and Electron Impact Mass Spectroscopy (EI MS) *m*/*z* (% rel. abund): 460 (M^+^, 1%).

Dioctyl phthalate (DOP): colorless liquid; ^1^H-NMR (500 MHz, CDCl_3_) 0.95 (t, J = 7.5 Hz, CH_3_), 1.30 (m, CH_2_), 1.39 (m, CH_2_), 1.69 (m, CH_2_), 4.22 (m, CH_2_), 7.53 (dd, J = 2–9 Hz, aromatic), 7.71 (dd, J = 2.5–9 Hz, aromatic); ^13^C-NMR (176 MHz, CDCl_3_) δ 14.1, 22.9, 23.8, 29.7, 30.3, 68.1, 128.8, 130.9, 132.4, 167.8; and EI MS *m*/*z* (% rel. abund): 390 (M^+^, 1%).

**Figure 9:**
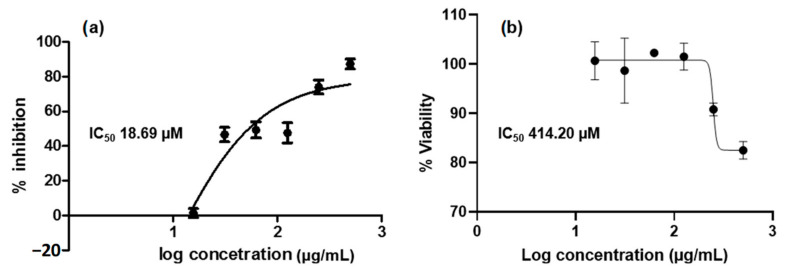
The drug-dose dependent curves of OTC against (**a**) MMP9 (IC_50_ 18.69 µM; R^2^ 0.8099) and (**b**) 4T1 cells (IC_50_ 414.20 µM; R^2^ 0.8473). NNGH was used as the positive control by showing IC_50_ 47.8 nM. The bars showed the standard error of triplicate assay as previously described.

The in vitro study revealed that OTC is active against MMP9 with IC_50_ 18.69 µM, whereas the IC_50_ of this compound in the 4T1 cell is 414.20 µM. The drug-dose dependent curve of OTC against MMP9 and 4T1 cells are illustrated in Figure 9. Unfortunately, neither MMP9 nor 4T1 cells were responsive toward DOP, confirming that this isolated compound from IC ethylacetate is not the one responsible for the activity in MMP9 and 4T1 cells as demonstrated by its original partition.

### 2.4. Molecular Docking

**Figure 10:**
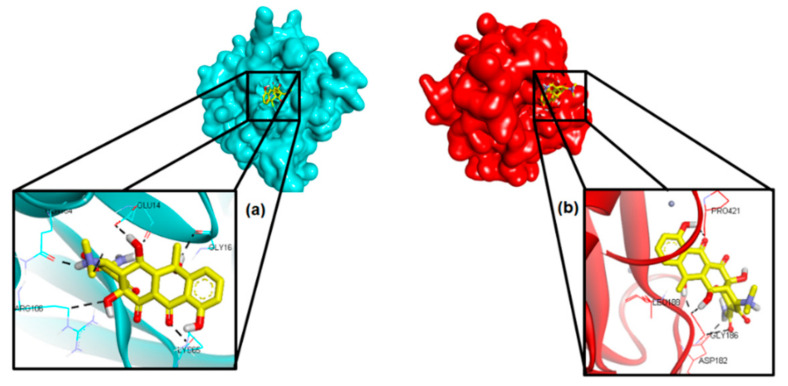
The docking poses OTC at the binding site of (**a**) PEX9 domain and (**b**) its catalytic domain.

To study the molecular mechanism on how OTC inhibits the activity of MMP9, molecular docking was performed on two domains of MMP9, the hemopexin (PEX9) domain and the catalytic domain. In our previous study [8], based on the preliminary study of virtual screening on the local plants, AC was selected due to the favorable binding of sesamin on the PEX9 domain of MMP9 [18]. However, instead of sesamin, we managed to isolate OTC, which was not present in our list of plant compounds to screen. Thus, it was interesting to also investigate the binding of OTC onto PEX9. Docking result showed that OTC bound well to PEX9’s active site with the free energy of binding (FEB) −7.59 kcal/mol. The OTC binding mode agreed with the previous study [4,19,20], suggesting that GLU14 and ARG106 are the most essential residues playing a key role in the PEX9 activity. In addition, OTC was also docked to the catalytic site of MMP9 and the FEB was −6.99 kcal/mol. This result indicates that OTC could bind to PEX9 more strongly than at its catalytic site. Figure 10 illustrates the binding of OTC to both the PEX9 domain as well as its catalytic domain.

### 2.5. GC-MS Profiles of the Partitions

**Figure 11:**
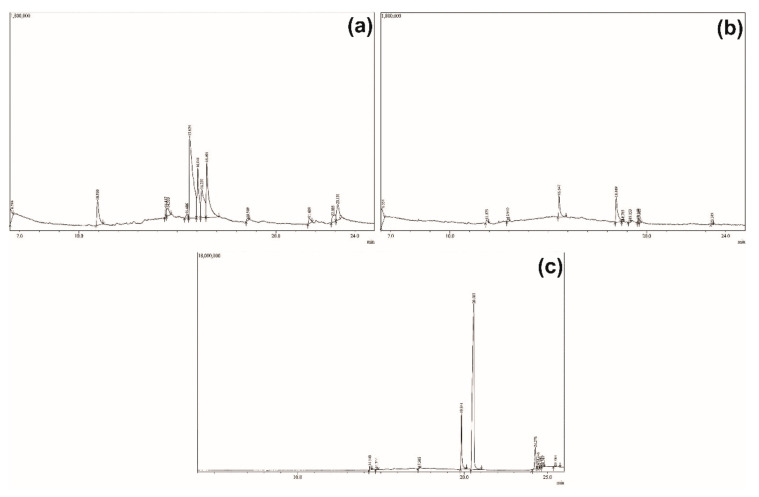
The GC chromatogram of (**a**) AC *n*-hexane, (**b**) IC *n*-hexane, and (**c**) IC ethylacetate, showing 10 to 13 peaks in a separable retention time. The highest intensity was detected at 15.624 min, 18.449 min, and 20.585 min for AC *n*-hexane, IC *n*-hexane, and IC ethylacetate partitions, respectively.

**Figure 12:**
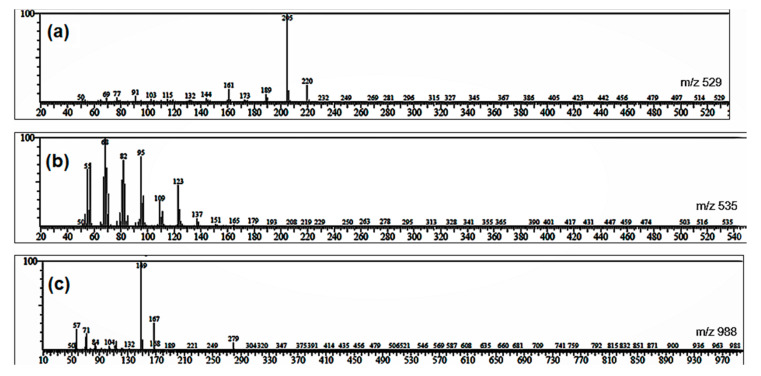
The mass spectra of three peaks identified from GC chromatogram of (**a**) AC *n*-hexane, (**b**) IC *n*-hexane, and (**c**) IC ethylacetate. The base peak informs the most stable fragment during electron impact in MS characterization.

Although there is only one compound able to be isolated from each AC and IC partition, we were also interested in probing other compounds that could be further identified from those plants using GC-MS (Figure 11 and Figure 12). In the AC *n*-hexane partition, there were 13 compounds detected by GC-MS. The compound with the highest intensity (R_t_ 15.624 min; 37.5%) was detected in *m*/*z* 529. To the best of our knowledge, this compound with its molecular weight has never been reported as presenting in AC, therefore, it needs further identification in future studies. IC *n*-hexane was detected as having 10 compounds with the one at 18.449 min showing the highest intensity (34.29%). This compound’s *m*/*z* was calculated as 535, and based on the literature, IC was reported as containing a compound with Molecular Weight (MW) 535 and characterized as ixorapeptide II. Ixorapeptide II is one of the markers in IC instead of ixorapeptide I (MW 500.6) [21]. Unfortunately, we were not successful in isolating this compound from the partition. On the other hand, the IC’s ethylacetate partition also detected the presence of 10 compounds with the one at 20.585 min showing the highest intensity (64.65%). This compound’s *m*/*z* was calculated as 988, with its molecular structure described as having a non-drug-like structure, in which the MW was usually around 500. This compound was predicted to be a longer ixorapeptide, but further experiment is warranted for confirmation.

## 3. Discussion

**Figure 13:**
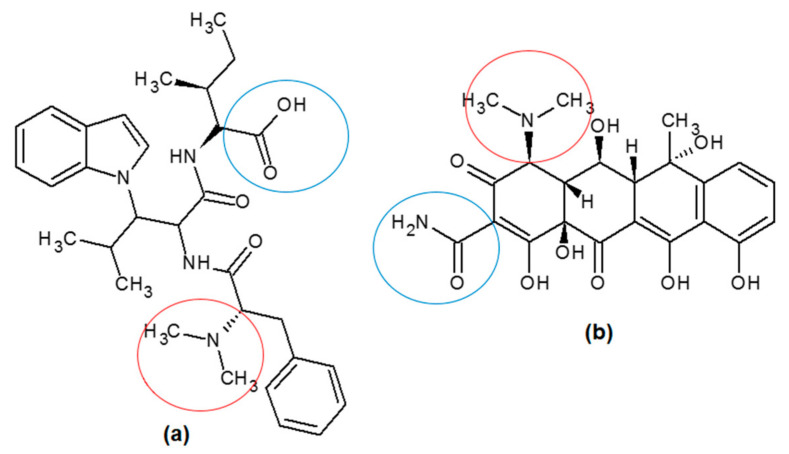
The structures of (**a**) ixorapeptide II and (**b**) OTC, which have a similar functional group, could be the responsible part of the compounds toward MMP9 inhibition.

Further exploration of AC and IC partitions demonstrated encouraging results, in which the IC ethylacetate partition showed potent activity against MMP9. This could be correlated with the compound identified during GC-MS analysis, ixorapeptide II, which had a peptidomimetic structure suitable for a protease inhibitor. Most likely, the proteolytic activity of MMP9 is dependent upon the presence of a fluorogenic peptide substrate. MMP9 has been shown to cleave Dnp-Pro-Leu-Gly-Met-Trp-Ser-Arg-OH at the Met-Trp sequence upon peptide hydrolysis that removes the N-terminal dinitrophenyl group [22]. Ixorapeptide II (Figure 13a) also had Trp bonded to the isopropyl side chain [23], which could act as a competitive inhibitor against the peptide substrate. Therefore, this compound could exert its activity at the MMP9 catalytic site rather than its PEX9 domain. This was in contrast with the AC *n*-hexane partition, in which OTC was the identified compound during the isolation process. OTC, being smaller compound than ixorapeptide II, had a structure that mimicked the peptide substrate less than ixorapeptide II did. According to the molecular docking study, OTC was suggested to prefer the PEX9 domain rather than its catalytic site. This, however, has a more promising anticancer potential due to the higher selective inhibition to MMP9 than other MMPs, leading to a safe drug. 

The cytotoxicity study showed that the IC ethylacetate partition was slightly more potent to both triple-negative breast cancer cell types (MDA-MB-231 and 4T1) than the AC *n*-hexane partition. Nonetheless, both partitions had potent concentrations in MDA-MB-231, which could be due to the MMP9 inhibition mechanism. Both partitions also showed potent inhibitions toward MCF7, which were predicted to have a dual mechanism. These observations were similar to other studies that showed inhibition of MMP9, as well as disruption of a hormonal-type cancer cell (Luminal A subtype) such as MCF7 [24,25]. However, the other Luminal A subtype, T47D, was not very responsive toward either partition. Thus, a plausible suggestion might be that both partitions are preferably useful for triple-negative cancer. With regards to the overall selectivity index, both partitions demonstrated high safety when applied to either triple-negative cancer or luminal A cancer cell types, with some exception for T47D, in which they showed a low selectivity index.

Another triple-negative type cell model from BALB/c mouse was observed as being not too responsive against the samples. It was understood that MMP-9 expressed by a metastatic cancer cell such as 4T1 could be the indicator of the cancer cell progression; therefore, the inhibition activity of MMP9 by compound (sample) should have corresponded to its cancer cell antiproliferation. However, the in vitro experiment toward the MMP9 enzyme here only evaluated how the sample inhibits such enzyme activity without considering its expression regulation by the cells. The fact of a less active sample against 4T1 cell proliferation than MMP9 activity could have been due to the direct inhibition only without taking down MMP9 expression by the cell. Further evaluation using zymography analysis is urgently needed to confirm whether MMP9 expression is affected by the samples [4].

Another probability is that the 4T1 cell was using the fifth generation, which could have been already resistant to the samples. This was observed by treating the 4T1 cells using doxorubicin, which was even less active than the plant samples. This could have been due to the increase in P-glycoprotein expression in 4T1 cells. This P-glycoprotein over-expression reduced doxorubicin uptake into the nucleus and caused the doxorubicin molecule to stay mostly in the cytoplasm. This was evidenced by the pre-treatment using verapamil (a P-glycoprotein inhibitor) upon 4T1 cell treated with doxorubicin, which may have reversed the chemo-resistance of this cell towards doxorubicin. Therefore, further evaluation by using verapamil pre-treatment is strongly recommended to confirm this effect [26]. The active compound’s metabolism into inactive form during cell penetration is also another possibility. 4T1 cells may express detoxifying genes such as TP53, which can alter the active form of the compound into inactive metabolite against 4T1 cell proliferation [27]. 

To our surprise, AC *n*-hexane partition led to the isolation of OTC. OTC is a class of aromatic polyketide antibiotic that is biosynthetically produced by a type II polyketide synthase that generates the poly-*β*-ketone backbone through successive decarboxylative condensation of malonyl-CoA extender units, followed by modifications by cyclases, oxygenases, transferases, and additional tailoring enzymes [28]. Antibiotics are known to be produced by bacteria, wherein OTC is originally produced by *Streptomyces rimosus*. Thus, it is questionable how a flowering plant was able to produce antibiotics, as normally they are produced by microorganisms. However, a study by Helfrich et al. (2018) [29] suggested that a wild plant also could produce antibiotics abundantly. They said that because of nutrient deficiencies, bacteria in the phyllosphere part of the plants (which lives above the ground) produce a diversity of substances that allows them to defend their habitat against other microorganisms. This was also confirmed by Prof. Vorholt, who identified more than 200 species of bacteria that live on the leaves of *Arabidopsis thaliana*. This plant is widely used as a model organism to provide a large library of genetic information that includes decoded genomes of the bacteria that colonize the plant’s leaf surfaces.

Intriguingly, OTC is an analog of doxycycline, a well-known antibiotic that also possesses MMP9 inhibitory activity in a very potent dose and lack of adverse side effects [30]. This drug, Periostat^®^, is the only MMP9 inhibitor that has passed phase three of clinical trials. However, the dose of Periostat^®^ is well tolerated while also improving the outcome when it is prescribed for periodontitis, not for cancer [31]. A study by Arnoczky et al. (2004) [32] indicated that OTC inhibits tractional structuring of collagen fibrils by equine myofibroblasts through an MMP-1 mediated mechanism. Fugler et al. [33] demonstrated the effect of lipopolysaccharide to enhance the MMP2 and MMP9 inhibitory activity of OTC in horse, resulting in OTC as the most effective drug inhibiting MMP2 and MMP9 compared to doxycycline.

Moving from OTC, we will now discuss the isolated compound from IC ethylacetate partition, DOP, which is usually detected as a contaminant from the plastic container during GC-MS analysis. However, we confirmed this compound was not a contaminant because the NMR of this isolated compound supported the GC-MS analysis. A study by Anusha et al. (2014) [34] reported that the *n*-hexane and ethylacetate partitions of IC contained diethyl phthalate and dibutyl phthalate [35]. This report, however, supported our result that the DOP we observed could have been originally from the plant, and was not a contaminant. Unfortunately, this compound showed no-inhibition toward both MMP9 or 4T1 and thus, the activity of both IC *n*-hexane and ethylacetate partitions toward MMP9 and cancer cells was not due to the presence of DOP, but rather of other compounds that to date, we have not successfully isolated.

Ixorapeptide II, which was previously identified in IC [21], was probably present in *n*-hexane partition indicated by the MS analysis. It may be more sensible that this compound was responsible for the IC *n*-hexane partition inhibition against MMP9. The structure of ixorapeptide II mimics a common protease inhibitor with a peptidomimetic structure. On the other hand, there were some functional groups in ixorapeptide II (*N*-dimethyl and carboxylate) that were similar to the ones in OTC (*N*-dimethyl and amide) (Figure 13). This might support our prediction that ixorapeptide II could be the compound presenting in IC *n*-hexane partition as having activity against MMP9. A study by Kini et al. (1995) [36] reported that another ixorapeptide (ixorapeptide I) from IC showed antiplatelet activity with IC_50_ of 29.52 µg/mL associated with MMP2 and MMP9 inhibitions [37].

There should be another way around studying the molecular mechanism of how the sample could stop metastatic cancer cell proliferation, instead of MMP9 FRET-based assay being established. Actin cytoskeleton reorganization enhances cell migration, contributing to cell invasion and metastasis in breast cancer. The protein that could be expressed during this step is Cdc42. To understand the insight mechanism on how this protein could interfere with this effect, we carried out in silico molecular docking of OTC and DOP against Cdc42 at the Guanosine Triphosphate (GTP)-binding site. The results showed that OTC (−5.64 kcal/mol) had higher FEB than DOP (−6.73 kcal/mol) while interacting with a GTP-binding site less associated with its affinity than DOP against cancer, which might be irrelevant based on the in vitro result. This may presume an irrelevant mechanism of OTC against cancer through actin cytoskeleton reorganization. These docking results can be seen in Figure S7 (Supplementary Material).

## 4. Materials and Methods

### 4.1. Software and Hardware

PEX9 1ITV [38] and MMP9 1GKC [39] were downloaded from the Protein Databank (PDB, www.rcsb.org (accessed on 2 March 2021); by the National Science Foundation (Alexandria, VA, USA), the US Department of Energy (Washington, WA, USA) and the National Cancer Institute (Bethesda, MD, USA), National Institute of Allergy and Infectious Diseases (Bethesda, MD, USA), and National Institute of General Medical Sciences of the National Institutes of Health (Bethesda, MD, USA)) accessed on 30 August 2020, and the 3D structure of OTC and DOP were downloaded from PubChem (pubchem.ncbi.nlm.nih.gov (accessed on 2 March 2021); National Center for Biotechnology Information (Bethesda, MD, USA)) accessed on 30 August 2020. The molecular docking used AutoDock1.5.6 and AutoDock4.2 and the output was visualized using Biovia Discovery Studio 2016 (www.accelrys.com (accessed on 2 March 2021); Dassault Systemes Biovia Corp. (San Diego, CA, USA)). An HP laptop with Core i3 processor on a Windows 10 operating system with 4 GB RAM and 500 GB Hard Disk was the hardware. 

### 4.2. Chemicals

The partitions (AC *n*-hexane, IC *n*-hexane, and IC ethylacetate), and the AC *n*-hexane-ethylacetate fraction were obtained from our previous study [8]. The HPLC grade of methanol, acetonitrile, and phosphate buffer pH 7.4 were used as the mobile phase, whereas the stationary phase used C_18_ column. The MMP9 enzyme kit was obtained from BioVision and comprised lyophilized MMP9, FRET-based MMP9 substrate (Mca-Pro-Leu-Gly-Leu-Dpa-Ala-Arg), MMP9 assay buffer, and NNGH inhibitor (*N*-isobutyl-*N*-(4-methoxyphenylsulfonyl)-glycyl hydroxamic acid) as its positive control. MDA-MB-231, MCF7, T47D, and 4T1 cells were obtained from Parasitology Laboratory, Medical Faculty, Gadjah Mada University), and cultured in Dulbecco’s Modified Eagle Media (DMEM) for MDA-MB-231, MCF7, and T47D, and RPMI-1640 medium containing 10% (*v*/*v*) fetal bovine serum (FBS) and 1% penicillin–streptomycin was used as the media for 4T1 (Life Technologies, Carlsbad, CA, USA) at 37 °C in a 5% CO_2_ humidified incubator. RNase was courtesy from Parasitology Laboratory, Medical Faculty, Gadjah Mada University and cultured in Dulbecco’s Modified Eagle Media (DMEM). Doxorubicin (DOX) and 3-(4,5-dimethylthiazol-zyl)-2,5-diphenyl tetrazolium bromide (MTT) were obtained from Sigma-Aldrich (St. Louis, MO, USA).

### 4.3. In Vitro MMP9 Inhibition Assay

The method of in vitro MMP9 inhibition assay referred to our previous publication [8]. The series of concentrations for the partitions were 31.25, 62.5, 125, 250, and 500 µg/mL, whereas the concentration for OTC was 62.5, 125, 250, 500, and 1000 µg/mL.

### 4.4. In Vitro Cytotoxicity Assay

The method of in vitro cytotoxicity assay is similar to our previous work [8]. The series of concentrations for the partitions and OTC were 15.625, 31.25, 62.5, 125, 250, and 500 µg/mL. The SEM imaging was carried by fixing the breast cancer cell being cultured in the 3D media, and in Karnovsky solution for 30 min at 4 °C. The media containing the cell was filtered out using millipore and then flushed with cacodylate 0.1% (3×), followed by dehydrating them using ethanol 96% and hexamethyldisilazane for 15 min. The whole cell was fixed on the stub, coated with 5 nm palladium gold film, and then visualized with Scanning Electron Microscope (SEM) (Quanta FEG 650; FEI Company (Hillsboro, OR, USA)) with a secondary electron mode [40]. 

### 4.5. Isolation

The AC *n*-hexane-ethylacetate fraction (200 mg) was dissolved in 10 mL of methanol and then injected into an HPLC instrument (Shimadzu SPDM20A; Shimadzu (Kyoto, Japan)) with the condition as follows: mobile phase methanol: acetonitrile: phosphate buffer pH 7.2 (70:27:3); flow rate 2.8 mL/min; temperature ambient; pressure 27; detector Photodiode Array (PDA). The resolved peak with the highest area was selected, separated, and then collected from the sample. The collected compound was then dried from its solvent and re-run through the same HPLC system to confirm it as a single peak (a pure compound).

On the other hand, the IC *n*-hexane-ethylacetate fraction was dissolved in *n*-hexane: ethylacetate (3:1) and spotted along the horizontal line on the prepared TLC plate. The plate was then developed in the Thin-Layer Chromatography TLC chamber containing *n*-hexane: ethylacetate (3:1) and then dried up. Upon drying, the plate was visualized under UV_254_ and UV_365_ illumination to mark some bands being resolved. The bands were then isolated and purified from the silica using methanol. The filtrate was then evaporated under reduced pressure to collect the pure single compound. The structural characterization of the isolates from AC and IC were performed using FTIR (Thermo Scientific Nicolet iS10), NMR (JNM-ECZ500R/S1), and GC-MS (QP2010S SHIMADZU).

### 4.6. Molecular Docking

The crystal structure of PEX9 with sulfate ion was downloaded from the Protein Data Bank (PDB) with PDB ID 1ITV. This ion was used as the native ligand and found located at blade 3 and blade 4 of the pocket site. PEX9 was presented as a homo-dimer in the crystal structure and in this study, only one monomer was used in the modeling. The sulfate ion was separated from PEX9 using Biovia Discovery Studio 2016, saved as a PDB file, and then assigned with Gasteiger Charges using AutoDockTools1.5.6 [41]. PEX9 was prepared using the same program whereby polar hydrogens were retained and the molecule was assigned with Kollman charges. The grid box was sized 70, 70, 70, with spacing 0.375 Å and center x = −42.05, y = −30.85, z = −7.26 and the docking was run 250× using AutoDock4.2 with the default parameters. On the other hand, the catalytic site validation was observed by docking *N*~2~-[(2*R*)-2-{[formyl(hydroxy)amino]methyl}-4-methylpentanoyl]-*N*,3-dimethyl-*L*-valinamide into 1GKC with the grid box size 60, 60, 60, spacing 0.375 Å, and center x = 65.607, y = 31.083, z = 117.843. The same docking parameter with PEX9 was applied here and the docking parameter was defined as valid, provided that the RMSD values of the complex were less than 2 Å [42]. 

## 5. Conclusions

The present study further supported our previous report about the potency of AC and IC as herbals for breast cancer remedies due to their positive action toward MMP9 and four cancer cells, including triple-negative sub-types (MDA-MB-231 and 4T1) as well as luminal A subtype (T47D and MCF7). Among three partitions, IC ethylacetate demonstrated the best IC_50_ against MMP9 (2.75 × 10^−2^ µg/mL) and 4T1 cells (57.5 µg/mL), followed by IC *n*-hexane (5.66 µg/mL (MMP9) and 225.5 µg/mL (4T1)) and AC *n*-hexane (246.1 µg/mL (MMP9) and 265 µg/mL (4T1)). On the other hand, the AC *n*-hexane partition showed the best activity against T47D (IC_50_ 109.70 µg/mL), among other partitions, i.e., IC ethylacetate (371.5 µg/mL) and IC *n*-hexane (1320 µg/mL). Furthermore, the cytotoxicity evaluation of MDA-MB-231 and MCF7 was also applied to AC *n*-hexane and IC ethylacetate partitions, resulting in a potent concentration (IC_50_ 1.92–2.11 µg/mL) to inhibit the cell proliferation for both partitions. The compounds that were suggested as responsible for the MMP9 inhibition as well as breast cancer cytotoxicities were oxytetracycline (OTC), with IC_50_ 18.69 µM and 414.20 µM, respectively. Unfortunately, dioctyl phthalate (DOP) isolated from the IC ethylacetate was inactive against both MMP9 and 4T1 cells. However, based on the GC-MS prediction, the compound that could be responsible for MMP9 and all cancerous cell proliferation was ixorapeptide II. The safety index measurements revealed that AC *n*-hexane and IC ethylacetate were selective toward MDA-MB-231 and MCF7, but toward 4T1, IC *n*-hexane, and IC ethylacetate they were the most selective cytotoxic agent.

## Figures and Tables

**Table 1:** The in vitro cytotoxicity assay results of the three partitions against MDA-MB-231, 4T1, T47D, MCF7 and Vero cell growth. IC_50_ here describes the concentration of extract that inhibits at least 50% cell proliferation.

	Samples	IC_50_ (µg/mL)				
		MDA-MB-231 (R^2^)	4T1 (R^2^)	T47D (R^2^)	MCF7 (R^2^)	Vero (R^2^)
1	AC n-hexane	2.05 (0.98)	265 (0.78)	109.70 (0.98)	2.11 (0.97)	217.2 (0.99)
2	IC n-hexane	NA	225.5(0.73)	1320 (0.86)	NA	972.2 (0.85)
3	IC ethylacetate	1.92 (0.99)	57.5 (0.85)	371.5 (0.95)	2.01 (0.98)	429.5 (0.99)
4	doxorubicin	NA	388.4 (0.52)	5.13 (0.91)	NA	69.58 (0.87)

NA = not applicable.

**Table 2:** The safety index (SI) of the three partitions is calculated from the ratio between the IC_50_ of the samples in the cancer cells over its IC_50_ in the Vero cells.

	Samples			SI	
		MDA-MB-231	4T1	T47D	MCF7
1	AC *n*-hexane	105.95	0.82	1.97	102.93
2	IC *n*-hexane	NA	4.31	0.74	NA
3	IC ethylacetate	223.69	7.47	1.16	213.68
4	doxorubicin	NA	0.18	13.56	NA

NA = not applicable.

## Data Availability

Not applicable.

## Supplementary Materials

The following are available online, Figure S1: The mass spectrum of OTC; Figure S2: The ^1^H-NMR spectrum of OTC; Figure S3: The FTIR spectrum of OTC; Figure S4: The ^1^H-NMR spectrum of DOP; Figure S5: The ^13^C-NMR spectrum of DOP; Figure S6: The mass spectrum of DOP; Figure S7: The overlapped pose of OTC and DOP into the GTP-binding site of Cdc42, Table S1: The additional information of the GC-MS condition.

## Sample Availability

Samples of the compounds are AC *n*-hexane, IC *n*-hexane and ethylacetate partitions available from the authors.
