# Supplementary material for: Bioguided Fractionation of Local Plants against Matrix Metalloproteinase9 and Its Cytotoxicity against Breast Cancer Cell Models: In Silico and In Vitro Study (Part II)
Source: Molecules. 2021 Mar 8;26(5):1464. doi: 10.3390/molecules26051464 (PMC7962846; doi:10.3390/molecules26051464)
Supplement: Supplementary file 1 [file molecules-26-01464-s001.pdf]

molecules

MDPI

Supplementary Materials

Bioguided Fractionation of Local Plants against Matrix Metalloproteinase9 and Its Cytotoxicity against Breast Cancer Cell Models: *In Silico* and *In Vitro* Study (II)

Maywan Hariono[1]*, Rollando Rollando[2], I Yoga[1], Abraham Harjono[1], Alfonsus Suryodanindro[1], Michael Yanuar[1], Thomas Gonzaga[1], Zet Parabang[1], Pandu Hariyono[1], Rifki Febriansah[3], Adi Hermawansyah[4], Wahyuning Setyani[1], and Habibah Wahab[5]*

1 Drug Discovery Student Club, Faculty of Pharmacy, Sanata Dharma University, Campus III, Paingan, Maguwoharjo, Depok, Sleman 55282, Yogyakarta, Indonesia; mhariono@usd.ac.id
2 Bachelor in Pharmacy Program, Faculty of Science and Technology, Ma Chung University, Malang 65151, Indonesia; ro.llando@machung.ac.id
3 School of Pharmacy, Faculty of Medicine and Health Sciences, Universitas Muhammadiyah Yogyakarta, Kasihan, Bantul 55183, Yogyakarta, Indonesia; rifki.febriansah@umy.ac.id
4 Cell Culture Laboratory, Faculty of Medicine and Health Sciences, Universitas Muhammadiyah Yogyakarta, Kasihan, Bantul 55183, Yogyakarta, Indonesia; hermawansyah_adi@yahoo.com
5 Pharmaceutical Technology Department, School of Pharmaceutical Sciences and USM-RIKEN Centre for Ageing Science (URICAS), Universiti Sains Malaysia, 11800 Minden, Pulau Pinang, Malaysia; habibahw@usm.my
* Correspondence: mhariono@usd.ac.id ; Tel.: +62-895-0628-6901

Figure S1: The mass spectrum of OTC.

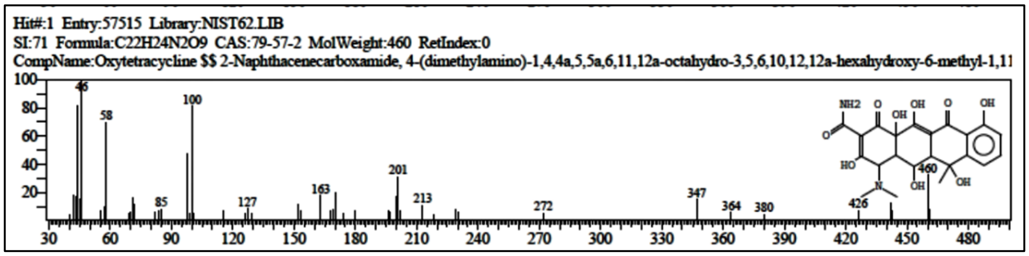

Figure S2: The ^{1}H-NMR spectrum of OTC.

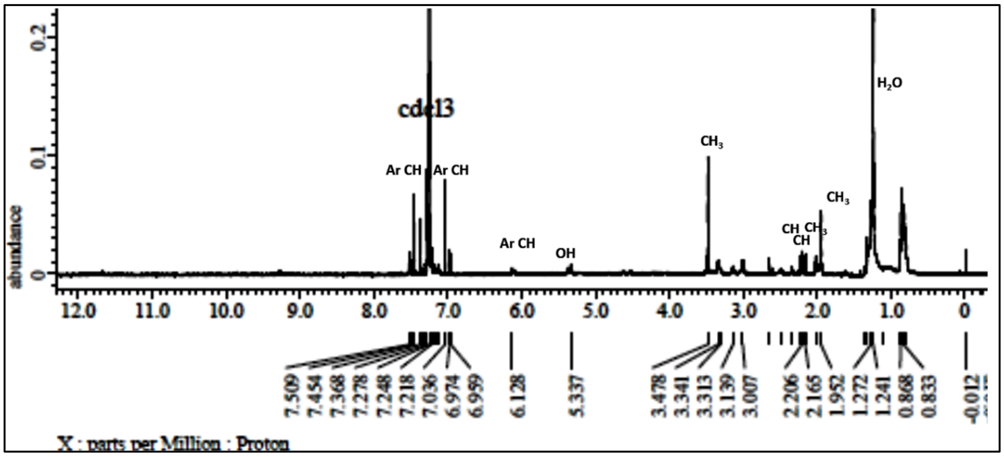

Figure S3: The FTIR spectrum of OTC.

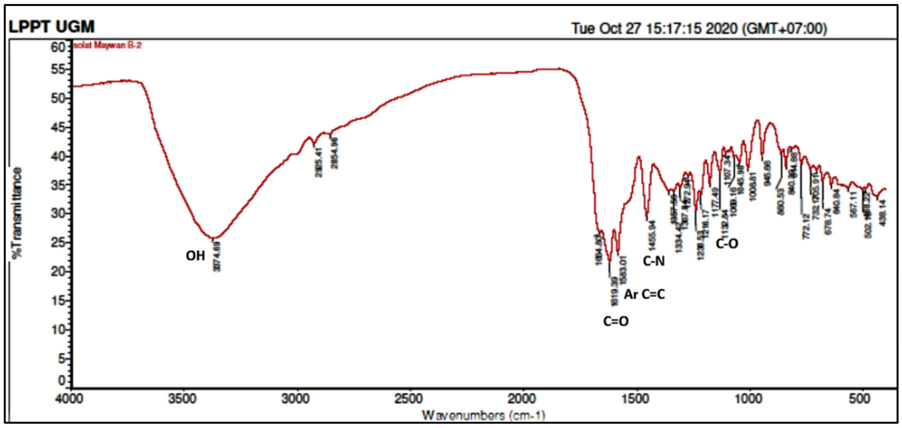

Figure S4: The ^{1}H-NMR spectrum of DOP.

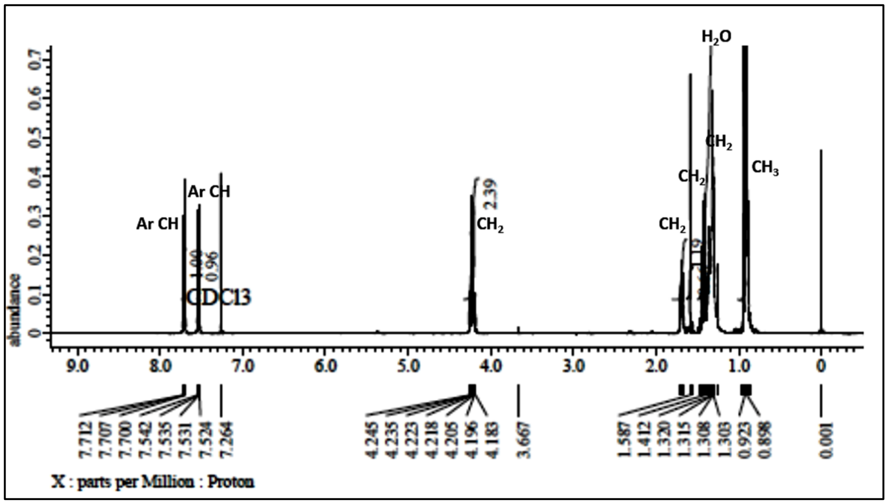

Figure S5: The ^{13}C-NMR spectrum of DOP.

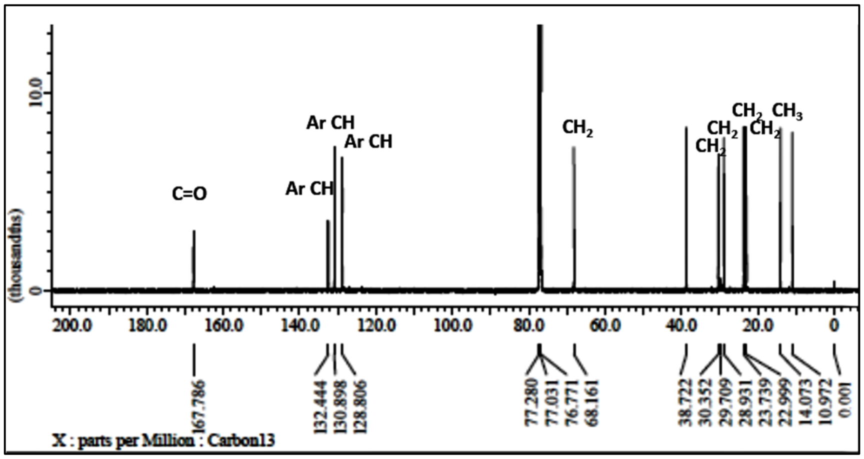

Figure S6. The mass spectrum of DOP.

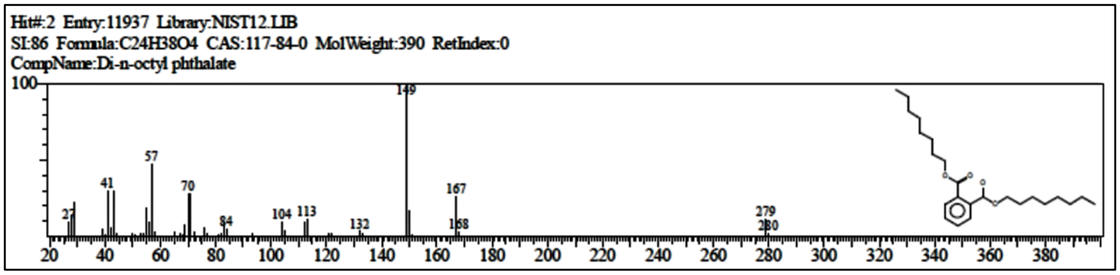

Figure S7. The overlapped pose of OTC and DOP into the GTP-binding site of Cdc42. The FEB of DOP = -6.73 kcal/mol whereas OTC = -5.64 kcal/mol. The protein is presented in surface model (orange), the ligands are presented in blue stick model (OTC) and green stick model (DOP).

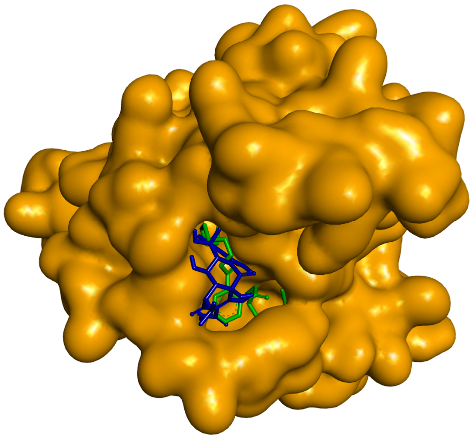

GC-MS condition :

Sample : 5 mg / 1 mL of chloroform

Volume of injection : 0.5 μL

Column : Rtx 5 MS column (diphenyldiethylpolysiloxane)

Carrier gas : Helium

Ion source temperature : 250 °C

Table S1. The additional information of the GC-MS condition.

Samples	Initial Temperature	Final Temperature	Rate	Held Time	m/z range
AC *n*-hexane	-	50 °C	-	2 mins	50 - 550
	50 °C	130 °C	20 °C/min	5 mins	
	130 °C	180 °C	12 °C/min	10 mins	
IC *n*-hexane	-	50 °C	-	2 mins	50 - 550
	50 °C	130 °C	20 °C/min	5 mins	
	130 °C	180 °C	12 °C/min	10 mins	
IC ethylacetate	-	60 °C	-	3.40 mins	50 - 1000
	60 °C	240 °C	12 °C/min	5 mins	
	240 °C	290 °C	60 °C/min	2 mins	
